# Supplementary material for: Low Concentration of the Neutrophil Proteases Cathepsin G, Cathepsin B, Proteinase-3 and Metalloproteinase-9 Induce Biofilm Formation in Non-Biofilm-Forming Staphylococcus epidermidis Isolates
Source: Int J Mol Sci. 2022 Apr 30;23(9):4992. doi: 10.3390/ijms23094992 (PMC9102557; doi:10.3390/ijms23094992)
Supplement: Supplementary file 1 [file ijms-23-04992-s001.zip › ijms-1679352-supplementary/Supp Figs.pdf]

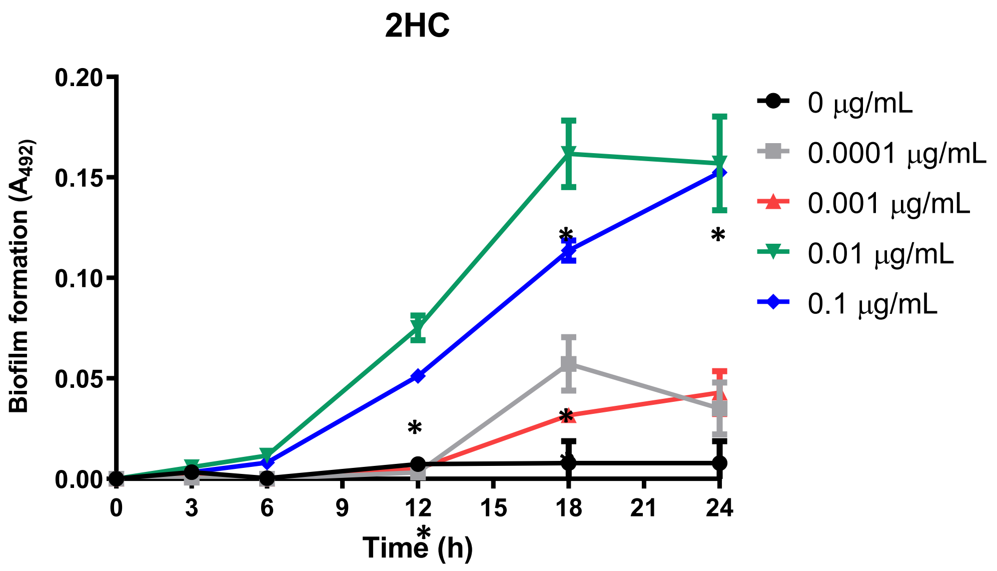


**Figure S1. Effect of cathepsin G in the biofilm formation kinetics of 2HC isolate.** Different concentrations of cathepsin G was added at the beginning of the biofilm formation kinetics of isolate 2HC (Healthy conjunctiva). The biofilm formation was determined at different times of culture by the method described by Christensen et. al. Asterisk * indicates a significant difference ($p < 0.05$) concerning the control (bacteria without cathepsin G). The statistical analysis was performed using a one-way ANOVA with a Tukey's test.

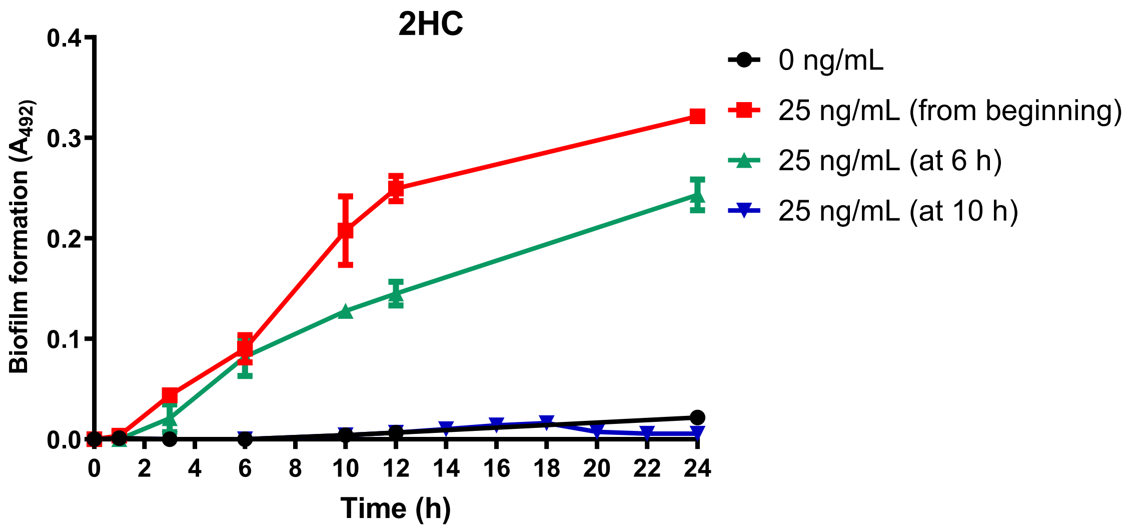


**Figure S2. Effect of proteinase-3 added at different stages of biofilm formation.** Biofilm forming kinetics of a commensal non-biofilm-forming isolate 2HC (healthy conjunctiva) was done in the presence of proteinase-3 added after 6 or 10 h from the starting point of culture. The abundance of biofilm was determined according to Christensen et al. sampling at different points of the kinetics. Asterisk * indicates a significant difference ($p<0.05$) concerning the bacteria with proteinase-3 from beginning. The statistical analysis was performed using a one-way ANOVA with a Tukey's test.

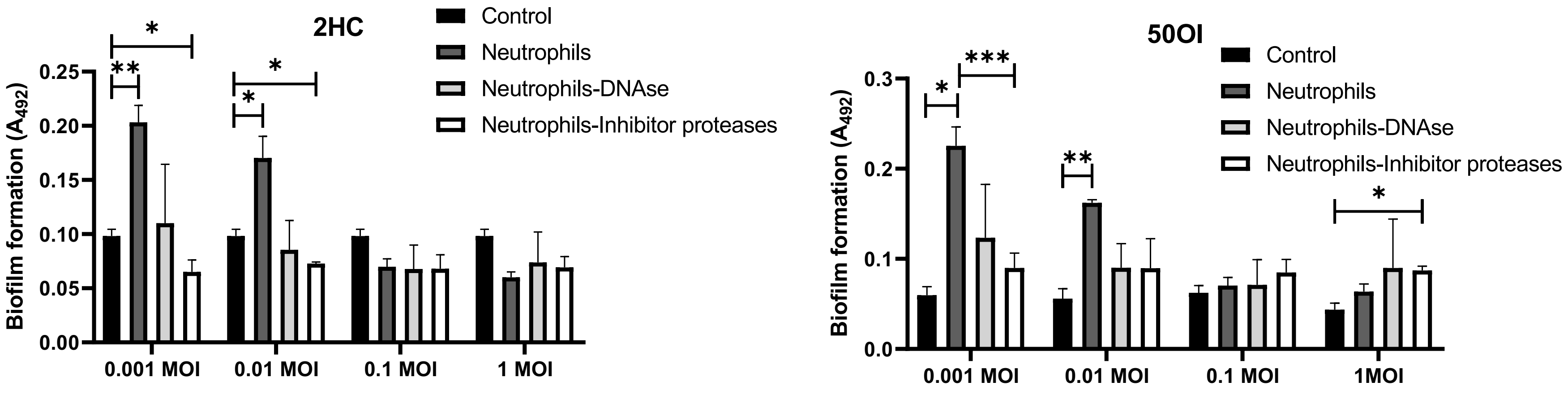


**Figure S3. Biofilm formation after DNAse treatment and proteinase-3 treatment**. Neutrophils (600 cells/mL) were cultured in the presence of different MOI of non-biofilm-forming isolates 2HC (Healthy conjunctiva; A) and 50 OI (Ocular infection; B) under different conditions: bacteria (control), neutrophils in the presence of bacteria without treatment (neutrophils), neutrophils with bacteria treated with 2 U DNAse I (Neutrophils-DNAse), neutrophils with bacteria treated with 1X cocktail of proteases inhibitors (Neutrophil-inhibitor proteases). After 24h of culture the biofilm formation was determinate by the method of Christensen et al. Asterisk * indicates a significant difference ($p < 0.05$). The statistical analysis was performed using a one-way ANOVA with a Tukey's test.

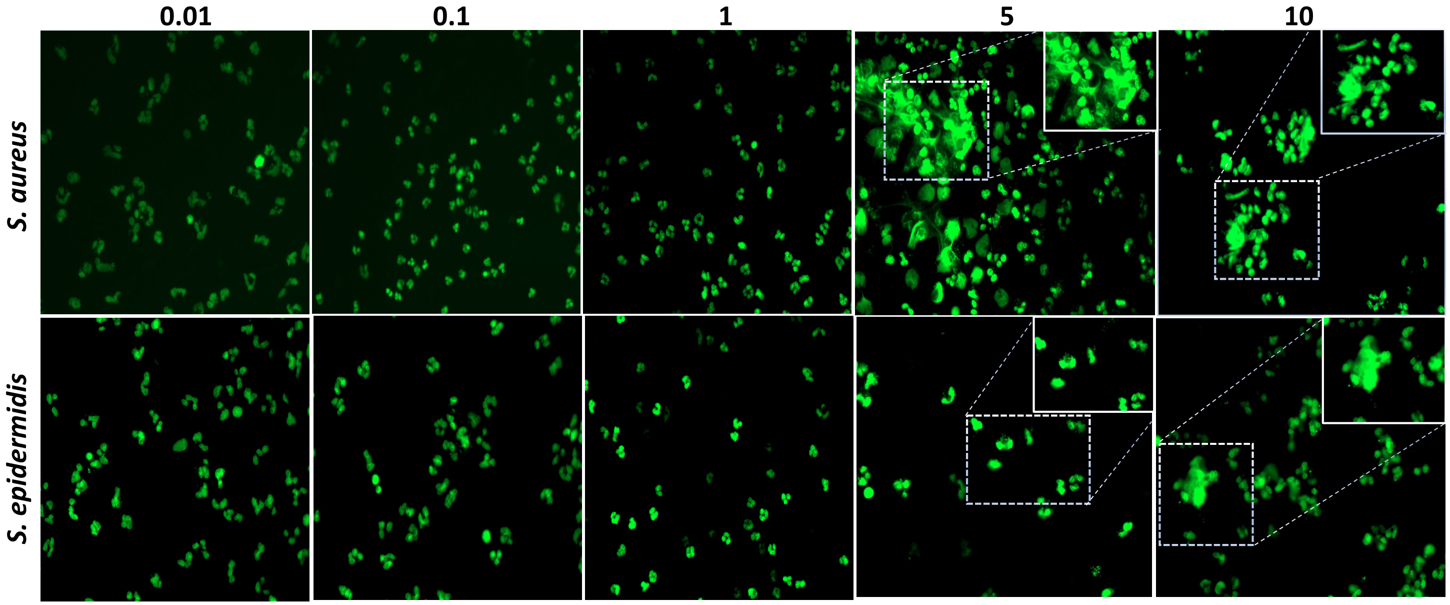


**Supplementary figure 4. NETs formation.** Neutrophils (600 cells/mL) cultured for 4 h in the presence of different MOI (0.01, 0.1, 1, 5 and 10) of non-biofilm-forming *S. epidermidis* 54HS isolate or *S. aureus* USA300 strains.

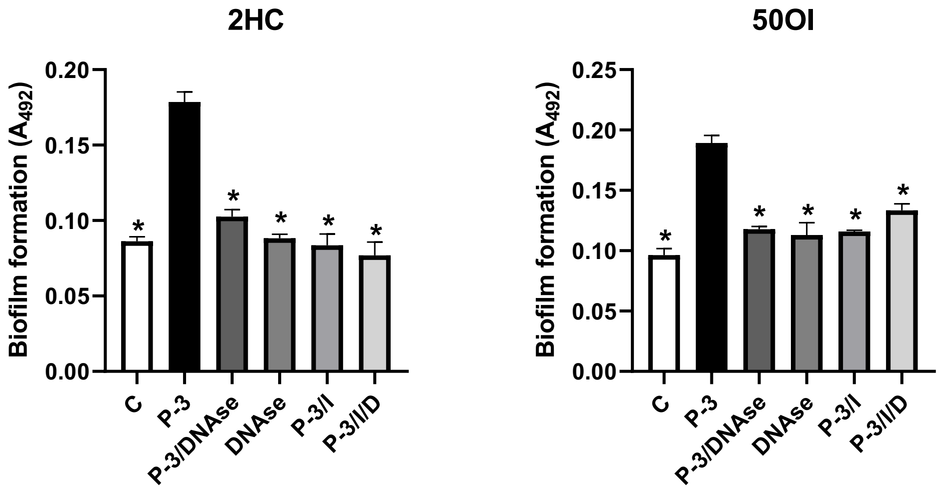


**Figure S5. Effect of DNAse in proteinase-3-induced biofilm.** Bacteria without treatment (control); bacteria treated with proteinase-3 (25 ng/mL) to form proteinase-3-induced biofilm (P-3); proteinase-3-induced biofilm treated with 2 U DNAse I (P-3/DNAse); bacteria treated with 2 U DNAse I (DNAse I); proteinase-3-induced biofilm treated with 5 mM PMSF inhibitor (P-3/I); proteinase-3-induced biofilm with 5 mM PMSF inhibitor more 2 U DNAse I (P-3/I//D). Isolate 2HC (A) and 50 OI (B). The abundance of biofilm was determined according to Christensen et al. Asterisk * indicates a significant difference ($p < 0.05$) to the P-3. The statistical analysis was performed using a one-way ANOVA with a Tukey's test.

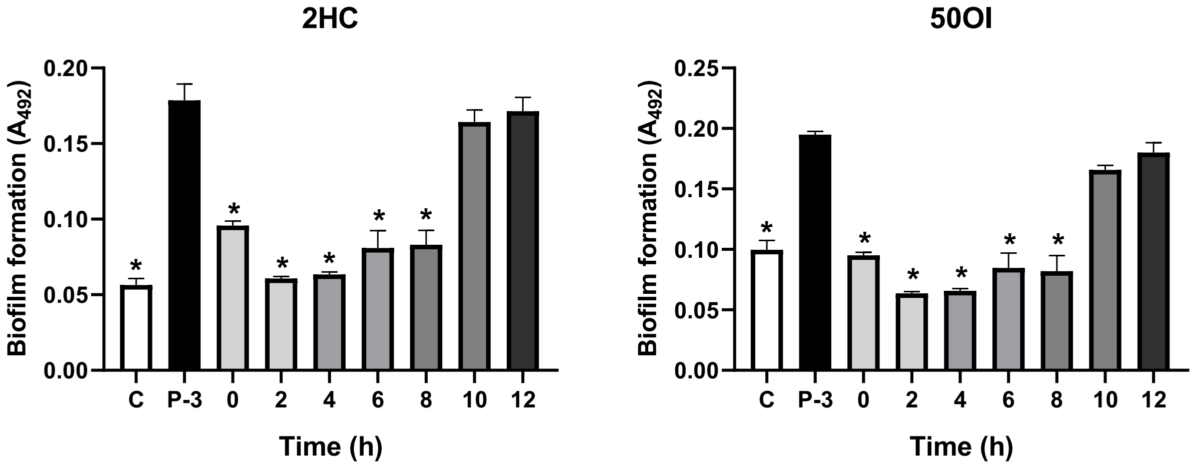


**Figure S6. Proteinase-3-induced biofilm treated with DNAse at different time.** Bacteria without treatment (C); bacteria treated with proteinase-3 (25 ng/mL) to form proteinase-3-induced biofilm (P-3); proteinase-3-induced biofilm treated with 2 U DNAse I at different times of culture (0, 2, 4, 6, 8, 10 and 12 h). Isolate 2HC (A) and 50 OI (B). The abundance of biofilm was determined according to Christensen et al. sampling at different times of culture. Asterisk * indicates a significant difference ($p < 0.05$) to the P-3. The statistical analysis was performed using a one-way ANOVA with a Tukey's test.
